# Antegrade Jejunojejunal Intussusception: An Unusual Complication Following Feeding Jejunostomy

**DOI:** 10.7759/cureus.13264

**Published:** 2021-02-10

**Authors:** Souradeep Dutta, Naveen Kumar Gaur, Abhinaya Reddy, Ankit Jain, Vishnu Prasad Nelamangala Ramakrishnaiah

**Affiliations:** 1 Surgery, Jawaharlal Institute of Postgraduate Medical Education and Research, Puducherry, IND

**Keywords:** jejunojejunal intussusception, intussusception, feeding jejunostomy, enteral nutrition, jejunostomy tube

## Abstract

Feeding jejunostomy (FJ) is a simple surgical procedure for enteral nutrition. But it can develop complications that may require re-exploration and can be life-threatening. Common complications include mechanical ones such as tube migration or dislocation, infection, gastrointestinal symptoms and fluid and electrolyte imbalances. However, intussusception is a rare complication of FJ. A 54-year-old gentleman underwent a D2 subtotal gastrectomy with Roux-en-Y gastrojejunostomy with FJ. On the sixth postoperative day, he developed severe colicky pain associated with abdominal distension and bilious vomiting. Ultrasonography and computed tomography revealed a 10-cm long jejunojejunal intussusception with the FJ tube at the center of the intussusception with proximal jejunal loops’ distension. The patient was taken up for a re-exploratory laparotomy with manual reduction of the intussusception and a new FJ insertion distal to the previous enterotomy site. The patient had an uneventful postoperative recovery.

## Introduction

Feeding jejunostomy (FJ) is a surgical procedure of inserting a tube in the proximal jejunum for enteral feeding. Enteral feeding is a preferable route for perioperative nutrition due to its trophic effects on the intestine causing less bacterial translocation and low infective complications. Considered a simple surgical procedure, FJ is often done by trainee surgeons. However, it is not free from complications, and some studies have quoted tube-related complications to be as high as 40%-50% [1]. Jejunojejunal intussusception is one such complication following it, rarely reported in the medical literature [2]. There are many methods described for FJ such as Stamms, longitudinal Witzel or transverse Witzel, but there are no relevant studies in the literature revealing the differences in complications rates between these techniques. Herein, we present the case of an elderly gentleman diagnosed with jejunojejunal intussusception following a gastrectomy with FJ.

## Case presentation

**Figure 1:**
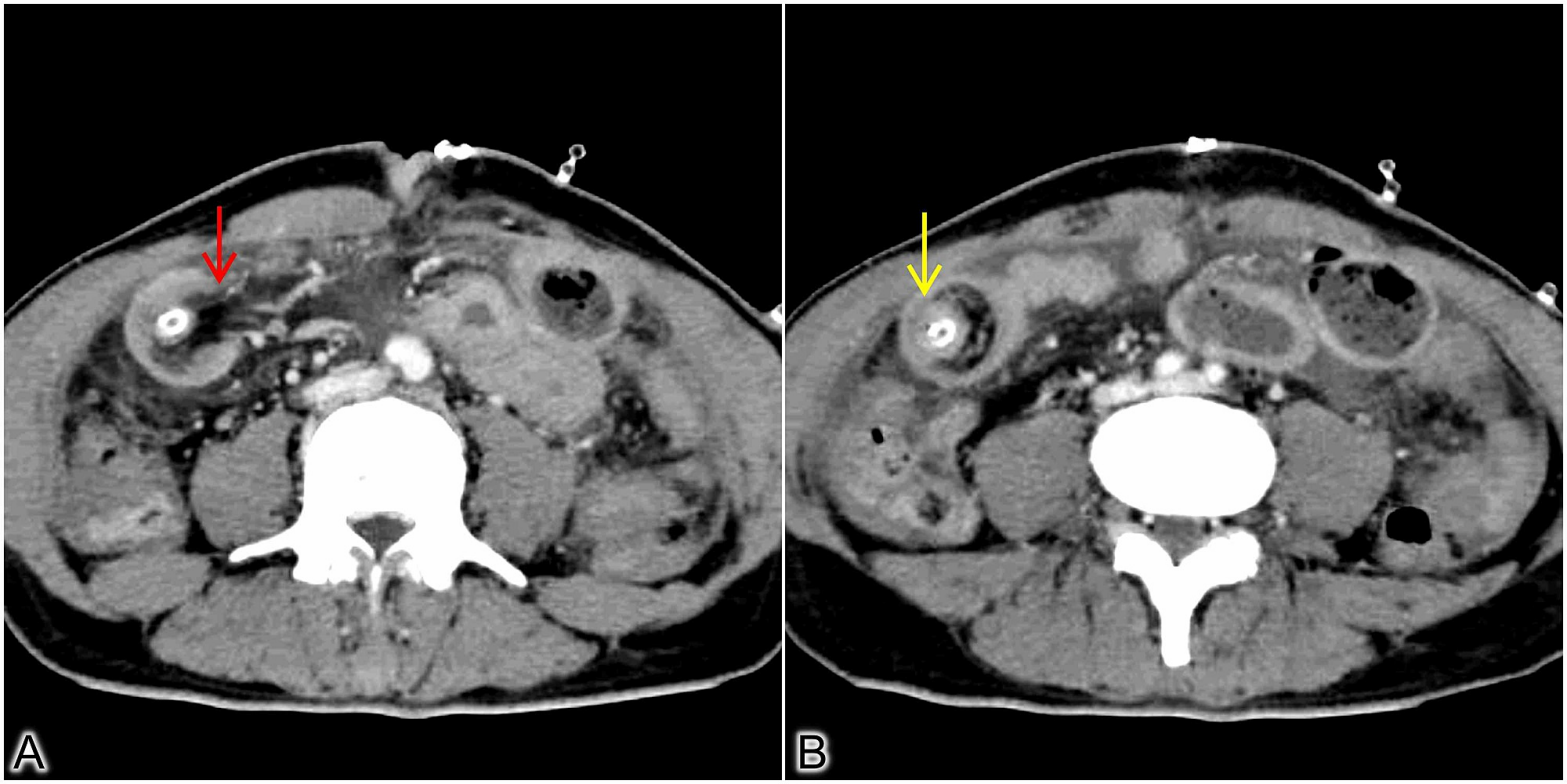
CECT images. (A) Axial section at the region of the start of intussusception. A bowel loop with its mesentery is seen entering into the adjacent bowel lumen with the tube in situ (red arrow). (B) Axial section at the mid of the intussusception showing the bowel within bowel appearance (yellow arrow). Bowel loops are seen well enhancing, indicating the intact vascularity. CECT - Contrast-Enhanced Computed Tomography

A 54-year-old gentleman presented with features suggestive of gastric outlet obstruction for 14 days. Upper gastrointestinal endoscopy revealed an ulceroproliferative growth at the antropyloric junction. He underwent a D2 subtotal gastrectomy with Roux-en-Y gastrojejunostomy with FJ using the Witzel technique following relevant imaging and other preoperative workups. Postoperatively, the patient was recovering well with adequate nutritional support through FJ. Oral liquids were started on the fourth postoperative day and gradually escalated. However, on the sixth postoperative day, he developed severe colicky pain associated with abdominal distension and bilious vomiting. An abdominal ultrasound was done, which showed bowel-in-bowel appearance in the jejunum with FJ tube within. Contrast-enhanced computed tomography (CECT) confirmed a 10-cm long jejunojejunal intussusception with FJ tube at the center of the intussusception with proximal jejunal loops’ distension (Figure 1).

**Figure 2:**
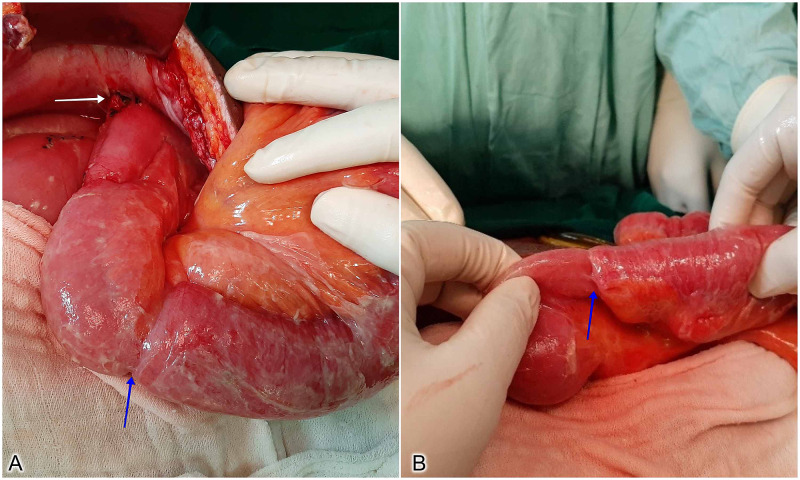
Intraoperative images showing the site of fixation of the jejunum to the parietal wall (white arrow), and the place where the jejunum is getting telescoped into its adjacent bowel’s lumen (blue arrows).

Because of persistent symptoms, the patient was taken up for re-exploratory laparotomy. Intraoperatively, a 12-cm long antegrade intussusception in the form of a fleshy sausage-like tubular intestinal mass was found at the proximal jejunum, 20 cm distal to the entry of the jejunostomy tube (Figure 2).

A careful complete manual reduction of the intussusception was performed. The bowel was edematous but healthy. The old FJ site was primarily closed, and a new FJ was done using Stamm’s technique, distal to the previous enterotomy site. Postoperative recovery was uneventful, and the patient was discharged two weeks later.

## Discussion

Jejunostomy is a surgical procedure for enteral feeding that is often done to improve the preoperative nutritional state in patients with a diseased upper digestive tract or as an additional procedure during a major upper gastrointestinal surgery if a complicated postoperative recovery is expected. There are many jejunostomy techniques, including open longitudinal Witzel, Stamm, needle catheter technique, percutaneous endoscopy and laparoscopy [3]. Common complications of FJ include mechanical complications such as tube migration/dislocation, infections (cutaneous or intra-abdominal abscesses), gastrointestinal symptoms (e.g., nausea, vomiting, diarrhea, constipation, abdominal distension), and fluid and electrolyte imbalances including hypokalemia, hypophosphatemia and hypomagnesemia [3]. Similar complication rates have been described for the two popular techniques - Witzel and Stamm. Intussusception is a rare complication of FJ. One series evaluating complications following jejunostomy tubes detected an intussusception rate of 1% in contrast radiographs [4]. This series was probably the first report of four jejunojejunal intussusception cases following jejunostomy tube placement, none of which required any surgical intervention and resolved with conservative management. Though further clinical information was not provided in this study, which could have favored a conservative management, most patients with long-segment intussusception and symptoms of intestinal obstruction, like in the current case, will require definitive surgical management.

Intussusception happens when a luminal irritant like an inflamed or edematous mucosa or a mass or polyp-like lesion acts as an obstructing lesion (lead point). This causes hyperperistalsis of the adjacent bowel resulting in the telescoping of a segment of the bowel, often with its mesentery to the adjoining distal bowel lumen [5]. Intussusception in adults is an uncommon entity accounting for 5% of all the cases of intussusceptions and 1% of small bowel obstruction [6]. Various postoperative factors that might lead to intussusception following FJ are adhesions around the suture lines, a long feeding tube, increased intra-abdominal pressure, shortening of the jejunal mesentery, and reverse peristalsis [7]. A large caliber of the jejunostomy tube or longer length of the tube placed within the bowel segment leads to distal tip migration, often acting as a lead point [8,9].

The diagnosis of FJ tube-induced intussusceptions is challenging as the clinical presentation is mostly nonspecific with no obstruction to the jejunostomy feeds. Moreover, the patients might be asymptomatic in 20% of cases [10]. A high degree of suspicion is required in patients with colicky abdomen pain with or without bilious vomiting on the fourth-fifth postoperative day following an FJ procedure [11]. Abdominal ultrasound is a quick useful bedside tool in the diagnosis of such an intussusception. The diagnostic features are the classical target and doughnut signs on the transverse view and a pseudo-kidney sign on a longitudinal view [12]. Though readily available without risk of radiation, it has limitations being operator dependent and technical limitations in certain situations such as obesity and increased bowel gas. A CECT of the abdomen is the most sensitive imaging modality to detect intussusception. Imaging features suggestive of intussusception are a target or sausage-shaped soft tissue mass enveloped within an eccentrically located area of low density, referring to a bowel within bowel configuration. Additionally, it can provide other important information such as length and diameter of the intussusception, possible lead point, the type and location of intussusception, the presence of strangulation or partial or complete bowel obstruction [13].

A short segment or a transient intussusception caused by FJ can be managed conservatively by changing the tube to a standard or short tube over a guidewire under fluoroscopic guidance or reduction by injecting air or contrast material through the tube [14]. However, cases with long segment persistent intussusception with features of partial or complete obstruction or features of strangulation require operative intervention. The definitive management is the surgical exploration and manual reduction, or resection of the intussusception segment based on intraoperative findings and bowel viability [15,16]. However, there is no single established management strategy, and the decision should be individualized for each patient as per the clinical scenario [17].

## Conclusions

A jejunojejunal intussusception is a rare complication following FJ. Clinical features are often nonspecific and require a high index of suspicion. An abdominal ultrasound can quickly detect intussusceptions; however, CECT is the most sensitive tool for diagnosing and detecting any complications. Though a conservative approach can be tried for simple transient cases, definitive surgical intervention is required for cases with obstruction or strangulation.
